# Molecular characterization of lumpy skin disease virus in Namibia, 2017

**DOI:** 10.1007/s00705-018-3891-x

**Published:** 2018-06-04

**Authors:** Umberto Molini, Gottlieb Aikukutu, Siegfried Khaiseb, Naindji N. Haindongo, Angela C. Lilungwe, Giovanni Cattoli, William G. Dundon, Charles E. Lamien

**Affiliations:** 1 Central Veterinary Laboratory (CVL), 24 Goethe Street, P. Bag 18137, Windhoek, Namibia; 2 Directorate of Veterinary Services, Luther Street, P. Bag 13184, Windhoek, Namibia; 3 Animal Production and Health Laboratory, Joint FAO/IAEA Division of Nuclear Techniques in Food and Agriculture, Department of Nuclear Sciences and Applications, International Atomic Energy Agency, Wagramer Strasse 5, P.O. Box 100, 1400 Vienna, Austria

## Abstract

Between January and July 2017, lumpy skin disease (LSD) outbreaks were reported in cattle in Namibia. DNA was extracted from skin biopsies taken from 32 cattle, and the RNA polymerase 30 kDa subunit (RPO30) gene of the LSD virus (LSDV) was successfully amplified by PCR. Phylogenetic analysis revealed that the newly sequenced LSDV isolates from Namibia were identical to LSDV isolates identified previously in Burkina Faso, Egypt, Greece, Niger, Serbia and South Africa. Given that only unvaccinated herds were affected by LSD, it is recommended that the current vaccination programmes in Namibia be re-evaluated to allow nationwide coverage.

Lumpy skin disease (LSD) is a disease of cattle caused by lumpy skin disease virus (LSDV), a DNA virus belonging to the genus *Capripoxvirus* within the family *Poxviridae* [3]. LSD was first reported in Zambia in 1929 from which it spread south to southern African countries and north to Sudan. The first diagnosis of LSD outside Africa was in Israel in 1989, followed by reports from Bahrain, Kuwait, Oman, Yemen, Lebanon, Jordan and Turkey [4, 11]. In 2015, LSD was detected in Europe (i.e., Greece and the Balkans) [1, 8]. LSD has a substantial economic impact in affected regions, causing decreases in milk yield, abortion and infertility in cows, and a decreased growth rate in beef cattle [9]. Morbidity rates can vary between 1 and 20%, although outbreaks with rates as high as 50% have been reported [4]. The control of LSD can be achieved through vaccination, restriction of animal movement, and culling of infected and exposed animals [2, 8]. Although LSD has been endemic in Namibia for many years, there is no genetic information available on local LSDV isolates. The present study describes the first genetic characterization of LSDV in the country.

During the period under investigation (January to July 2017), there were 32 LSDV outbreaks affecting 10 out of the 14 regions of Namibia (Fig. 1): Omaeke (4 outbreaks), Otjozondjupa (4 outbreaks), Hardap (2 outbreaks), Oshikoto (2 outbreaks), Kunene (9 outbreaks), Zambesi (2 outbreaks), Oshana (1 outbreak), Ohangwena (1 outbreak), Karas (1 outbreak), and Erongo (6 outbreaks). One representative sample from each outbreak was included in this study. Skin nodule biopsies were collected aseptically from sick cattle, and the samples were sent, refrigerated, to the Central Veterinary Laboratory (CVL) of Windhoek. Upon arrival at the CVL, the samples were stored at – 20 °C until processing. DNA was extracted from the tissue homogenates using a Maxwell^®^16 Tissue DNA Purification Kit (Promega, Madison, WI, USA) with an elution volume of 300 μl following the manufacturer’s instructions. A commercial real-time PCR assay, Genesig^®^ Advance Kit LSDV116 RNA polymerase subunit (Primerdesign^tm^ Ltd, Chandler’s Ford, UK), was used to confirm the presence of capripoxvirus DNA in all of the 32 samples tested, with Ct values between 24 and 36 being recorded. Next, two pairs of primers CpRPO30-OL1F (5’-CAGCTGTTTGTTTACATTTGATTTTT-3’) and CpRPO30-OL1R (5’-TCGTATAGAAACAAGCCTTTAATAGA-3’) (pair 1), CpRPO30-OL2F (5’-TTTGAACACATTTTATTCCAAAAAG-3’) and CpRPO30-OL2R (5’-AACCTACATGCATAAACAGAAGC-3’) (pair 2) were used to amplify two overlapping fragments of the RPO30 gene to generate the full-length sequence of the gene [5]. (Molecular epidemiological analysis using the RPO30 gene is used to genotype capripoxviruses. The variability within each genotype is generally very low, so any nucleotide sequence variation is highly indicative of a true difference between isolates, i.e., vaccine strains versus field isolates). The PCR was conducted in a reaction volume of 25 µl containing 500 nM forward primer, 500 nM reverse primer, 0.2 mM dNTPs, 1x PCR buffer (QIAGEN), 2.5 U of Taq polymerase (QIAGEN) and 5 µl of template DNA. An initial denaturation at 95 °C for 4 min was followed by 40 cycles at 95 °C for 30 s, 55 °C for 30 s, and 72 °C for 45 s, and then a final extension at 72 °C for 7 min. The expected positive PCR products of 554 bp (for pair 1 primers) and 520 bp (for pair 2 primers) were visualized on a 1.5% agarose gel for all of the 32 samples. The amplicons were purified using a Wizard SV Gel and PCR Clean-Up System (Promega) and sequenced commercially by LGC Genomics (Berlin, Germany). The RPO30 sequences from the 32 samples were edited and assembled using the Staden software package version 2.0.0b8. All of the generated sequences were submitted to GenBank with accession numbers MG757462 to MG757493. Multiple sequence alignments were performed using the ClustalW algorithm implemented in the BioEdit software package version 7.2.6 to compare the RPO30 gene sequences of the isolates involved in the outbreaks. Additional RPO30 gene sequences were retrieved from GenBank and included in the data set. For construction of phylogenetic trees, the neighbor-joining method in MEGA7 was used with the maximum composite likelihood nucleotide substitution model, the pairwise deletion option, and 1000 bootstrap replicates [6] (Table 1).

**Fig. 1:**
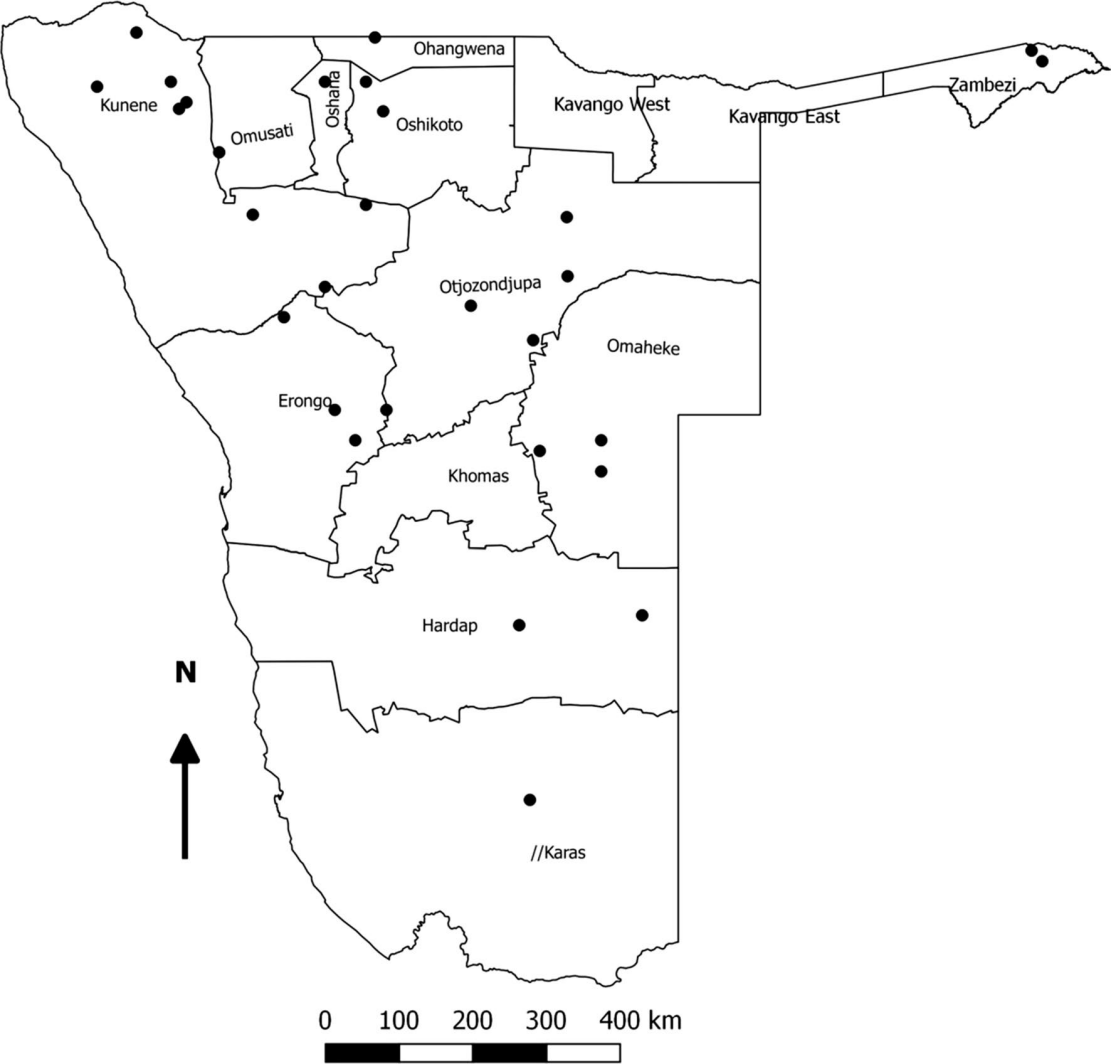
Geographic distribution of lumpy skin disease outbreaks reported in cattle in Namibia from January to July 2017

**Table 1:** RPO30 sequences submitted to GenBank

Sample ID	Accession number (GenBank)	Collection date	Place collected	Host
LSDV_NAM_30_1	MG757462	January 2017	Omaeke	Cattle
LSDV_NAM_30_2	MG757463	January 2017	Omaeke	Cattle
LSDV_NAM_211	MG757464	January 2017	Otjozondjupa	Cattle
LSDV_NAM_212	MG757465	January 2017	Otjozondjupa	Cattle
LSDV_NAM_415	MG757466	February 2017	Otjozondjupa	Cattle
LSDV_NAM_416	MG757467	February 2017	Otjozondjupa	Cattle
LSDV_NAM_871	MG757468	March 2017	Hardap	Cattle
LSDV_NAM_872	MG757469	March 2017	Oshikoto	Cattle
LSDV_NAM_941	MG757470	March 2017	Kunene	Cattle
LSDV_NAM_1001	MG757471	March 2017	Oshana	Cattle
LSDV_NAM_1169	MG757472	March 2017	Oshikoto	Cattle
LSDV_NAM_1172	MG757473	March 2017	Ohangwena	Cattle
LSDV_NAM_1429	MG757474	April 2017	Omaeke	Cattle
LSDV_NAM_1467	MG757475	April 2017	Zambesi	Cattle
LSDV_NAM_1468	MG757476	April 2017	Zambesi	Cattle
LSDV_NAM_1689	MG757477	April 2017	Kunene	Cattle
LSDV_NAM_1690	MG757478	April 2017	Kunene	Cattle
LSDV_NAM_1691	MG757479	April 2017	Kunene	Cattle
LSDV_NAM_1701	MG757480	April 2017	Omaeke	Cattle
LSDV_NAM_2190	MG757481	May 2017	Erongo	Cattle
LSDV_NAM_2191_1	MG757482	May 2017	Erongo	Cattle
LSDV_NAM_2191_2	MG757483	May 2017	Erongo	Cattle
LSDV_NAM_2191_3	MG757484	May 2017	Erongo	Cattle
LSDV_NAM_2226	MG757485	May 2017	Erongo	Cattle
LSDV_NAM_2229	MG757486	May 2017	Erongo	Cattle
LSDV_NAM_2864	MG757487	June 2017	Kunene	Cattle
LSDV_NAM_3166	MG757488	June 2017	Karas	Cattle
LSDV_NAM_3303	MG757489	June 2017	Kunene	Cattle
LSDV_NAM_3306	MG757490	June 2017	Kunene	Cattle
LSDV_NAM_3469	MG757491	June 2017	Hardap	Cattle
LSDV_NAM_3540	MG757492	June 2017	Kunene	Cattle
LSDV_NAM_3742	MG757493	June 2017	Kunene	Cattle

From the phylogenetic analysis of the RPO30 gene, it can be clearly seen that all of the biopsy samples collected in Namibia contained LSDV DNA and that the sequences were identical to each other (Fig. 2). In addition, they were identical to previously identified LSDVs from several countries both in Africa (Burkina Faso, Egypt, Niger and South Africa) and Europe (Greece and Serbia). Importantly, the RPO30 gene sequences of the Namibian field isolates were clearly different from those of the LSDV vaccines used in this country. In Namibia, three commercial vaccines are used, two of which contain cell-adapted strains of the so-called Neethling strain (Fig. 2, KX764644 and KX764645), and the third, an attenuated South African LSDV field isolate (Fig. 2, KX764643) [11]. It is possible that the LSDVs identified in Namibia and those in neighbouring South Africa share a common origin, but in order to confirm this, full-genome sequencing and further molecular epidemiological studies in the region are required. A recent characterization of the P32 gene of LSD samples in Zimbabwe identified two genetically distinct viruses circulating in the country [7]. A similar comparative study should be undertaken for other LSDV isolates collected in southern Africa in order to get a clearer picture of virus circulation at a regional level.

**Fig. 2:**
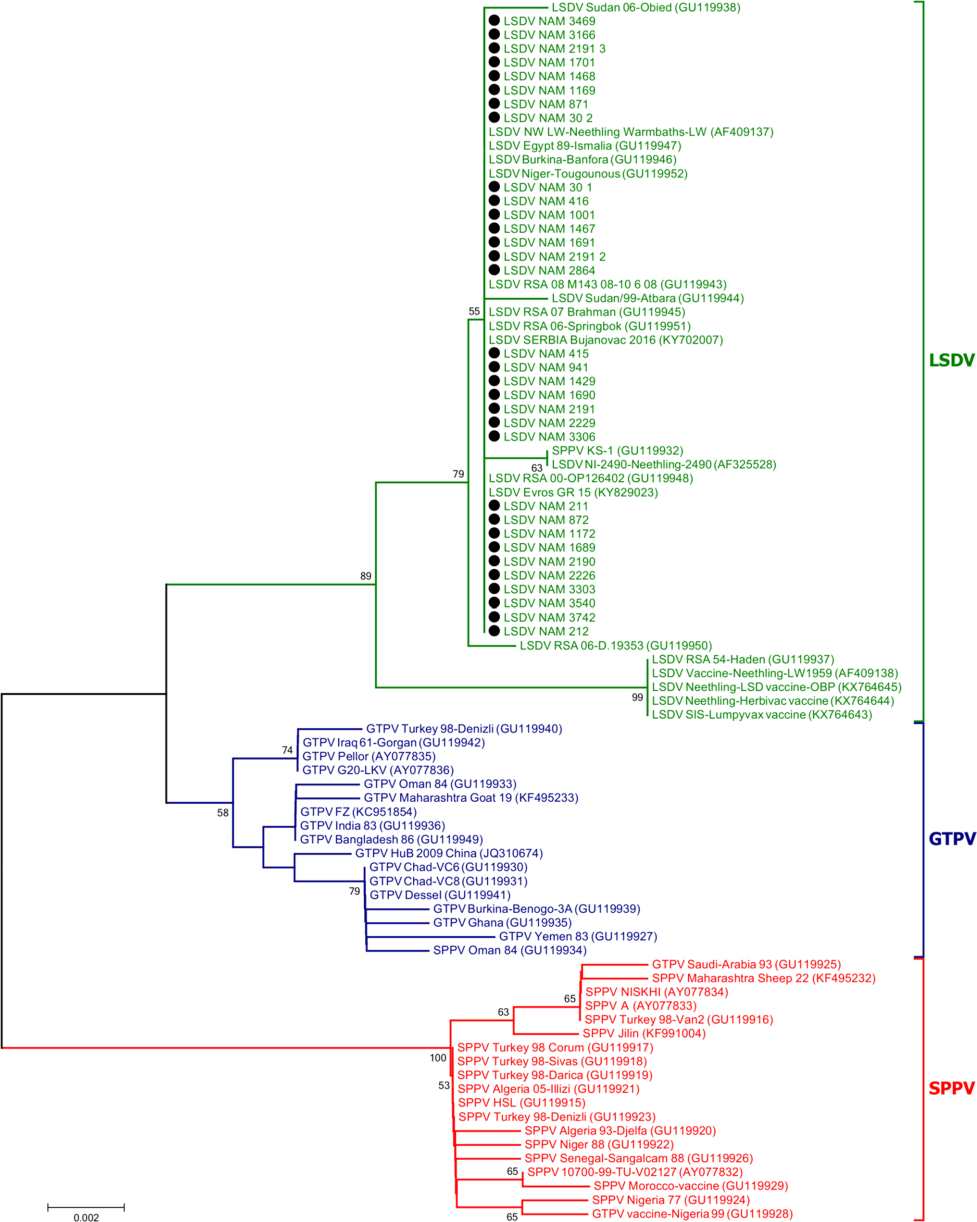
Thirty-two complete sequences of the LSDV RPO30 gene aligned and analyzed by neighbour joining using MEGA7 software [6]. Numbers indicate the bootstrap values calculated from 1000 bootstrap replicates. Black dots correspond to the newly sequenced samples from Namibia (accession numbers MG757462 to MG757493). LSDV stands for lumpy skin disease virus, SPPV for sheep pox virus, and GTPV for goat pox virus

This study has confirmed that LSDV is still present and distributed across the whole country despite vaccination programmes. Importantly, it was observed that only unvaccinated herds were affected by the disease, which suggests that the present vaccination strategy in Namibia requires reevaluation to improve coverage and participation by farmers. It is known that the use of live attenuated vaccines is an effective way to control the spread of LSD, although good protection can only be achieved if sufficient herd immunity (over 80%) is maintained by carrying out annual vaccinations [10].
